# GPER/PKA-Dependent Enhancement of Hormone-Sensitive Lipase Phosphorylation in 3T3-L1 Adipocytes by Piceatannol

**DOI:** 10.3390/nu16010038

**Published:** 2023-12-21

**Authors:** Kotoko Arisawa, Ayumi Matsuoka, Natsuki Ozawa, Tomoko Ishikawa, Ikuyo Ichi, Yoko Fujiwara

**Affiliations:** 1 Graduate School of Pharmaceutical Sciences, Tohoku University, Sendai 980-8577, Japan; kotoko.arisawa.b3@tohoku.ac.jp; 2 Graduate School of Humanities and Sciences, Ochanomizu University, Tokyo 112-8610, Japan; ayucolocollon1112@gmail.com (A.M.); g2240523@edu.cc.ocha.ac.jp (N.O.); 3 Institute for Human Life Science, Ochanomizu University, Tokyo 112-8610, Japan; ishikawa.tomoko@wa.seitoku.ac.jp (T.I.); ichi.ikuyo@ocha.ac.jp (I.I.); 4 Department of Human Nutrition, Seitoku University, Chiba 271-8555, Japan; 5 Natural Science Division, Faculty of Core Research, Ochanomizu University, Tokyo 112-8610, Japan

**Keywords:** piceatannol, hormone-sensitive lipase, adipocyte, lipolysis, G protein-coupled estrogen receptor (GPER)

## Abstract

We previously reported that piceatannol (PIC) had an anti-obesity effect only in ovariectomized (OVX) postmenopausal obesity mice. PIC was found to induce the phosphorylation of hormone-sensitive lipase (pHSL) in OVX mice. To elucidate the mechanism by which PIC activates HSL, we investigated the effect of PIC using 3T3-L1 adipocytes. PIC induced HSL phosphorylation at Ser563 in 3T3-L1 cells, as in vivo experiments showed. pHSL (Ser563) is believed to be activated through the β-adrenergic receptor (β-AR) and protein kinase A (PKA) pathways; however, the addition of a selective inhibitor of β-AR did not inhibit the effect of PIC. The addition of a PKA inhibitor with PIC blocked pHSL (Ser563), suggesting that the effects are mediated by PKA in a different pathway than β-AR. The addition of G15, a selective inhibitor of the G protein-coupled estrogen receptor (GPER), reduced the activation of HSL by PIC. Furthermore, PIC inhibited insulin signaling and did not induce pHSL (Ser565), which represents its inactive form. These results suggest that PIC acts as a phytoestrogen and phosphorylates HSL through a novel pathway that activates GPER and its downstream PKA, which may be one of the inhibitory actions of PIC on fat accumulation in estrogen deficiency.

## 1. Introduction

Estrogen is important for the protection of women’s health, since women are prone to various problems after menopause, such as osteoporosis, abnormal lipid metabolism, hypertension, and glucose intolerance [1,2,3]. It is also well known that postmenopausal women with decreased estrogen tend to gain body weight and fat mass [4,5]. Both male and female estrogen receptor α (ERα) and ERβ knockout mice showed significant levels of obesity and glucose intolerance [2,6], although their energy intake had not changed, suggesting that estrogen is important for regulating fat accumulation and energy balance in the entire body. One proposed explanation for the anti-obesity effect is that energy expenditure is reduced during estrogen deficiency [7,8]. ERα is the predominant modulator of estrogenic effects that regulate the size and number of adipocytes in adipose tissue [6]. Estrogen replacement therapy (ERT) has been shown to be effective in reducing menopausal obesity in human clinical studies. However, continuous use raises concerns due to various potential side effects, including increased risks of breast cancer and thrombosis. Therefore, it is imperative to explore alternative treatments [9,10,11].

Piceatannol (PIC, 3′,4′,3,5-tetrahydroxy stilbene), a structurally related analog of resveratrol (RES, 3,4,5′-trihydroxy stilbene), is a natural compound found in high concentrations in passion fruit (Passiflora edulis) seeds [12]. As with RES, PIC is known to have beneficial effects, including estrogenic, anti-inflammatory, antioxidant, and anticancer actions [13,14,15]. PIC has also been reported to activate sirtuin1 (SIRT1) [16]. One study, using a rat model, demonstrated that oral absorption of PIC was superior to that of RES and that its metabolites were more stable in plasma than those of RES [17]. Although there are fewer studies on PIC than on RES, especially in vivo, the research thus far suggests that PIC may be more effective than RES.

We previously reported that the addition of PIC to a high-fat diet inhibited body weight gain and visceral fat accumulation in female ovariectomized mice (OVX) but not in sham-operated mice [18]. PIC activated the uncoupling protein 1 (UCP1) in brown adipose tissue in OVX and sham mice, possibly by mediated SIRT1 activation by PIC. We also found that hormone-sensitive lipase (HSL) phosphorylation in white adipose tissue was extremely low in OVX mice and that PIC restored HSL activation. This is a specific effect of PIC that is observed only during estrogen deficiency.

HSL is, like adipose triglyceride lipase (ATGL), an enzyme that breaks down triglycerides (TG) stored in adipose tissue into fatty acids to provide an energy source in the blood in response to the body’s energy needs. HSL is activated through the β-adrenergic receptor (β-AR) and protein kinase A (PKA) pathway [19]. The phosphorylation of HSL by PKA facilitates its translocation from the cytosol to the surface of lipid droplets (LDs), where it cooperates with ATGL and other lipid-droplet associated proteins [20,21]. HSL mainly catalyzes diacylglycerol (DG), and the degraded free fatty acids (FFA) are released into the bloodstream.

Estrogen is recognized for its ability to downregulate lipogenic genes in adipocytes, the liver, and skeletal muscle through classical ERα genomic action as a nuclear receptor. However, there is growing interest in its non-genomic actions [22]. The G protein-coupled estrogen receptor (GPER) plays a role in mediating non-genomic, rapid signaling responses to estrogen [23]. GPER is involved in several intracellular signaling pathways, in addition to adenylyl cyclase and the PKA pathway, including the phosphatidylinositol 3-kinase (PI3K)-Akt and mitogen-activated protein kinase (MAPK) pathways, which exert cell growth, NOS production, and anti-inflammatory effects [24,25,26]. Therefore, GPER is recognized as a new target for estrogen-related diseases such as breast cancer, cardiovascular disease, and diabetes [27,28]. GPER knockout mice showed the development of pathological conditions such as obesity, dyslipidemia, and insulin resistance in males as well as females [29]. Studies with 3T3-L1 adipocytes suggest that GPER activation inhibits adipogenesis by disrupting mitotic clonal expansion [30].

RES and phytoestrogens, such as genistein and daidzein, are suggested to act as GPER agonists [31]. However, there are no reports that estrogen promotes HSL phosphorylation by PKA downstream of GPER signaling.

Therefore, to explore the mechanism by which PIC activates HSL during estrogen deprivation in mature adipocytes, we examined the effects of PIC using 3T3-L1 adipocytes.

## 2. Materials and Methods

### 2.1. Materials

Piceatannol was obtained from Tokyo Chemical Industry Co., Ltd. (TCI, Tokyo, Japan). Propranolol (Sigma-Aldrich, St. Louis, MO, USA) was used for β-AR signaling experiments. H89, a PKA inhibitor, was obtained from Sigma-Aldrich, and G15 (GRB-G15), a selective GPER antagonist, was purchased from Selleck Biotech (Tokyo, Japan). These reagents were dissolved in dimethyl sulfoxide (DMSO) and added to the culture medium for each experiment. All other reagents were of the highest commercial analytical grade.

### 2.2. Cell Cultures

3T3-L1 preadipocytes were obtained from the American Type Culture Collection (ATCC), seeded at 1 × 10^5^ cells/well into 6-well plates and cultured in Dulbecco’s modified Eagle medium high glucose (DMEM, Sigma-Aldrich, D6429) containing 10% fetal bovine serum (FBS, BioWest, Rennes, France) and 1% penicillin streptomycin (P/S, FUJIFILM Wako Pure Chemical Corporation, Osaka, Japan). After 2 days of confluency, 3T3-L1 preadipocytes were induced by adipogenic agents (0.5 mM 3-isobutyl-1-methylxanthine, 0.25 μM dexamethasone, and 10 μg/mL insulin) in DMEM containing 10% FBS (day 0). After 48 h of induction, the medium was changed to differentiation medium, which consisted of DMEM containing 10% FBS, 1% P/S and 10 μg/mL insulin. This change was made again every other day. On day 6 from the start of induction, mature adipocytes were used for the experiments. Conditions for the PIC treatment of the cells are described in the figure legends. For inhibitor experiments, adipocytes were preincubated with each inhibitor for 60 min and then incubated with 25 µM PIC for another 60 min, after which cells were harvested.

### 2.3. Western Blotting

The Western blotting procedure was performed using methods previously described [18]. Briefly, proteins were extracted from cells using a buffer containing a protease inhibitor cocktail (P8340; Sigma-Aldrich) and phosphatase inhibitor cocktails (P5726 and P0044; Sigma-Aldrich). After measurement of protein concentrations using the BCA assay (FUJIFILM Wako Pure Chemical Corporation), equivalent amounts of proteins were separated by 8% SDS-PAGE and transferred to polyvinylidene difluoride (PVDF) membranes (Bio-Rad Laboratories, Inc., Hercules, CA, USA). After blocking treatment (5% *w*/*v* of non-fat dry milk in Tris buffered saline containing 0.1% Tween-20, for 1 h at room temperature), membranes were incubated overnight at 4 °C with the following primary antibodies (all Cell Signaling Technology, Danvers, MA, USA): rabbit anti-phospho-HSL (Ser563) (CST#4139), rabbit anti-phospho-HSL (Ser565) (CST#4137), rabbit anti-HSL (CST#18381), rabbit anti-Akt (CST #9272), rabbit anti-phospho-Akt (Ser473) (CST #9272) and rabbit anti-β-Actin (CST#8457). The membranes were then incubated with peroxidase-conjugated anti-rabbit antibodies (Jackson ImmunoResearch Laboratories, West Grove, PA, USA; 111-035-144). Immunoreactivity was detected using ECL Prime Western blot detection reagents (Cytiva, Tokyo, Japan), with β-Actin serving as the loading control. The molecular weight marker used was the 3-Color prestained XL-ladder (APRO Science, Tokushima, Japan).

### 2.4. Oil-Red O Staining

Cells in a 6-well plate were washed twice with phosphate-buffered saline (PBS (−)) and fixed with 10% formalin. Intracellular LDs were stained with 0.2% oil-red O solution (Sigma-Aldrich) and microscopically examined.

### 2.5. Measurement of Intracellular Triglyceride Concentration

After washing with PBS (−), the adipocytes were scraped with 2 mL of methanol and the total lipid was extracted using the Bligh and Dyer method [32]. The extracted and dried lipid was dissolved in 50 µL isopropanol, and the triglyceride concentration was measured by an enzymatic method (Labo AssayTM Triglyceride, FUJIFILM Wako Pure Chemical Corporation).

### 2.6. Statistics

All data are presented as mean ± standard deviation (M ± SD). Statistical analysis was performed using Microsoft Excel for Mac version 16.70 and one-way analysis of variance (ANOVA) was performed using GraphPad Prism 8 software (GraphPad Software Inc., San Diego, CA, USA), followed by Student’s *t*-test. A *p*-value below 0.05 was considered statistically significant.

## 3. Results

### 3.1. Inhibition Effects of PIC on Fat Accumulation in 3T3-L1 Adipocytes

**Figure 1:**
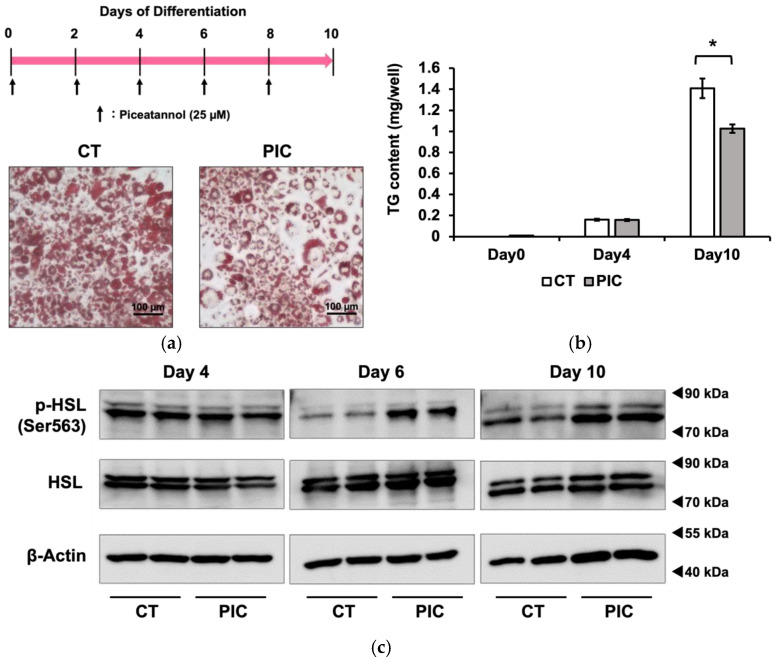
Continuous administration of piceatannol suppresses fat accumulation in 3T3-L1 adipocytes. To induce adipocyte differentiation, 3T3-L1 cells were treated with insulin, IBMX, and dexamethasone. PIC (25 µM) was added to cells every other day, commencing at the beginning of differentiation and continuing for a period of 10 days. (**a**) Oil Red O staining was used to visualize and evaluate lipid droplets in 3T3-L1 cells. (**b**) Intracellular triacylglycerol levels were quantified in 3T3-L1 cells on days 0, 4, and 10. (**c**) A Western blotting analysis was performed to measure pHSL expression in 3T3-L1 cells on days 4, 6, and 10, with β-Actin serving as a loading control. We included the Oil Red O staining on day 6 in 3T3-L1 cells as Figure S1. Data are presented as mean values ± standard deviation (*n* = 3 for each group). Statistical significance was assessed using a *t*-test. The asterisks (*) indicate significant differences between the control and PIC groups. These experiments were performed three times, which confirmed the reproducibility of the results.

We investigated the inhibitory effects of PIC on fat accumulation using 3T3-L1 adipocytes. The differentiation of 3T3-L1 cells into adipocytes was induced with IBMX, dexamethasone and insulin 2 days after confluence was reached. On day 2, the medium with insulin was replaced every other day. PIC was added during differentiation induction and freshly added during each medium change. After 10 days of differentiation induction, intracellular LDs in 3T3-L1 cells were stained with Oil Red O (Figure 1a). The results showed a suppression of fat accumulation in the PIC-treated group, particularly in the reduction in small to medium LDs. Subsequently, the intracellular triacylglycerol content was measured. There was no significant difference between the two groups on day 4, but on day 10, the PIC-treated group exhibited inhibition of fat accumulation (Figure 1b).

Previously, we reported that PIC reduced visceral fat accumulation in estrogen-deficient postmenopausal obese mice. We attributed this effect to PIC’s activation of hormone-sensitive lipase (HSL) through phosphorylation (pHSL) at Ser563, facilitating the lipolysis of TG within LDs in white adipose tissue [18]. Therefore, we investigated whether PIC could enhance pHSL (Ser563) in 3T3-L1 cells. As a result, we observed an increase in pHSL (Ser563) after PIC treatment on day 6 and day 10 of differentiation induction in 3T3-L1 cells (Figure 1c).

### 3.2. The Phosphorylation of HSL by PIC Is Regulated Independently of β-Adrenergic Receptors

Previous studies have found that PIC influences mitotic clonal expansion (MCE) in 3T3-L1 cells and reduces adipogenesis by inhibiting differentiation into adipocytes. However, our results in OVX mice suggest that the promotion of fat decomposition also plays a role in suppressing fat accumulation. Therefore, we conducted further research on the effects of PIC on fat accumulation in 3T3-L1 adipocytes that had accumulated fat after differentiation.

**Figure 2:**
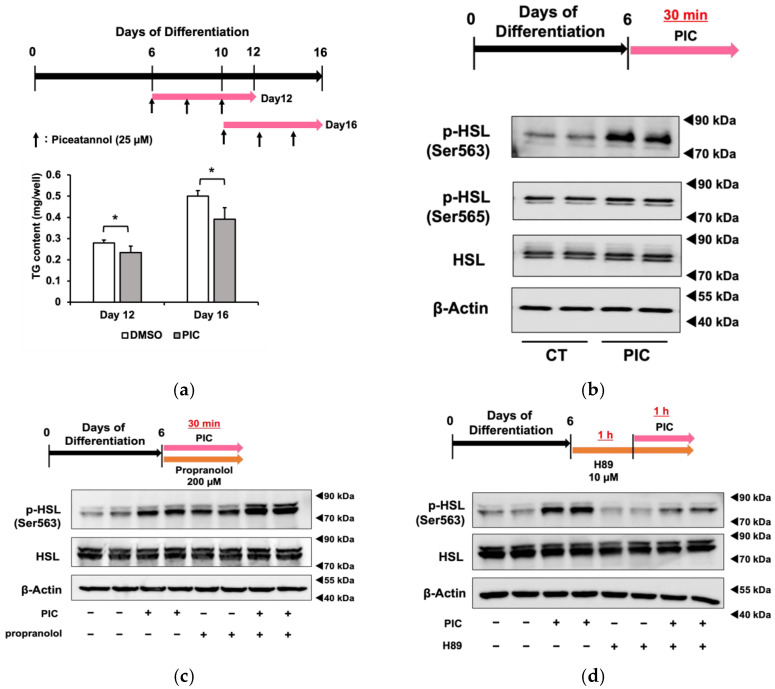
Piceatannol administration after adipocyte differentiation suppresses fat accumulation in 3T3-L1 cells. (**a**) After differentiating 3T3-L1 adipocytes for 6 or 10 days without the addition of PIC, 25 µM PIC was added for an additional 6 days. Intracellular triacylglycerol levels were quantified in 3T3-L1 cells on days 12 and 16. (**b**) On day 6, 3T3-L1 cells were treated with PIC for 30 min. The phosphorylation of HSL at Ser563 and Ser565 was evaluated by Western blotting. (**c**) 200 µM of the β-AR antagonist propranolol was added to the cells simultaneously with PIC and incubated for 30 min. (**d**) The PKA inhibitor H89 was pretreated one hour before the addition of PIC, and the expression level of HSL phosphorylation was assessed one hour after PIC was assessed. Data are presented as mean values ± standard deviation (*n* = 3 for each group). Statistical significance was assessed using a *t*-test. The asterisks (*) indicate significant differences between the control and PIC groups. These experiments were performed three times, which confirmed the reproducibility of the results.

In Figure 2a, we added PIC for 6 days beginning on day 6 or day 10 after the initiation of differentiation and measured triacylglycerol accumulation. The results demonstrated that PIC had an inhibitory effect on fat accumulation in differentiated 3T3-L1 adipocytes. Although PIC was indicated to inhibit adipocyte differentiation factors that are up-regulated in the early stages of differentiation, such as PPARγ, C/EBPα, β, and δ, our results suggested that the inhibition of fat accumulation by PIC after adipocyte differentiation could occur through pathways independent of these factors.

When PIC was administered for a short duration (30 min) to 3T3-L1 cells on day 6 of differentiation, HSL phosphorylation was enhanced in Ser563, similar to the results obtained with continuous treatment from differentiation initiation (Figure 2c). On the other hand, PIC did not change phosphorylation at the Ser565 site. HSL is a lipase that provides energy by lipolysis during times of energy deficiency. In fasting conditions, catecholamines such as adrenaline bind to β3-adrenergic receptors on the cell membrane in response to a decrease in blood glucose levels. This binding activates downstream adenylate cyclase, leading to the production of cAMP from ATP. As intracellular cAMP levels increase, protein kinase A (PKA) is activated and phosphorylates HSL at Ser563. Phosphorylated HSL (Ser563) then accesses LD in white adipocytes, where it cleaves ester bonds in triacylglycerol molecules, releasing fatty acids into the bloodstream for use as an energy source. The Ser565 phosphorylation site of HSL is phosphorylated by another pathway, and access to LD is, in contrast, inhibited [33]. Therefore, we investigated whether HLS phosphorylation by PIC depended on β-AR by employing the β-AR inhibitor propranolol (Figure 2c). The increase in pHSL (Ser563) due to PIC was not inhibited when propranolol was used to block β-AR, suggesting that PIC induces HSL phosphorylation independently of β-AR. We verified the efficacy of positive control experiments involving the addition of the β-AR agonist isoproterenol, which led to an increase in pHSL (Ser563). Furthermore, we found that 200 µM propranolol demonstrated sufficient inhibitory effects.

Subsequently, we used the PKA inhibitor H89 to determine whether PIC-induced HSL phosphorylation involved PKA (Figure 2d). In cells pretreated with H89 followed by the addition of PIC, we observed a reduction in HSL phosphorylation, indicating that PKA mediated the induction of pHSL (Ser563) by PIC.

These results suggested that PIC activates PKA without affecting β-AR and increases HSL phosphorylation.

### 3.3. Piceatannol Phosphorylates HSL via the GPER Pathway

Next, we focus on the G protein-coupled estrogen receptor (GPER) as an alternative factor involved in PKA activation, apart from β-AR. GPER differs in signaling mechanisms from classical estrogen receptors (ERs), such as ERα and ERβ. While ERα and ERβ are nuclear receptors, GPER is known to transmit signals through pathways involving second messengers within seconds to minutes of stimulation. Prior studies have mainly associated GPER with stimulating PKA activation through Gα subunit protein-mediated adenylate cyclase activation, leading to the proliferation of estrogen-sensitive cancer cells [34]. However, no previous reports have linked GPER to HSL phosphorylation in adipocytes.

**Figure 3:**
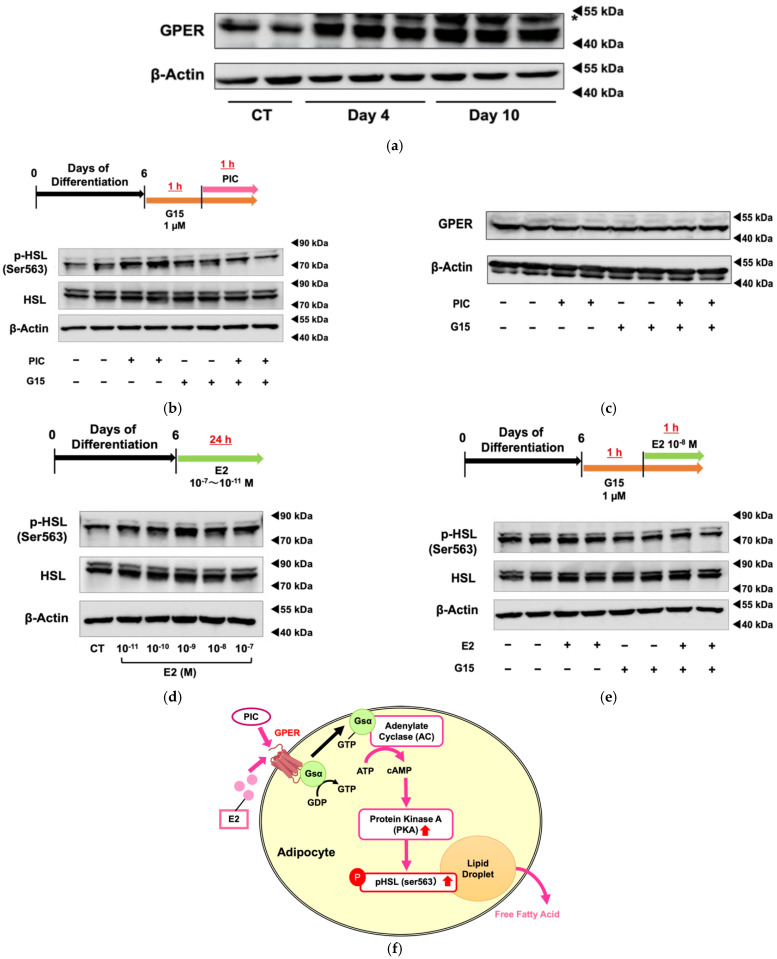
Piceatannol promotes HSL phosphorylation and lipolysis via GPER. (**a**) 3T3-L1 cells were induced to differentiate without the addition of PIC, and the protein expression level of GPER was evaluated on days 12 and 16. The band indicated by an asterisk is a nonspecific band. (**b**) For 3T3-L1 adipocytes on day 6, the specific GPER antagonist G15 was pretreated one hour before PIC addition. One hour after the level of addition of 25 µM PIC, the protein expression of phosphorylated HSL was evaluated. (**c**) The level of GPER was also evaluated. (**d**) On day 6, the 3T3-L1 adipocytes were exposed to estradiol (E2) at concentrations ranging from 10^−7^ to 10^−11^ M for 24 h, and the level of protein expression level of phosphorylated HSL was evaluated. (**e**) For 3T3-L1 adipocytes on day 6, G15 was pretreated one hour before the addition of E2. One hour after the addition of E2, the protein expression level of phosphorylated HSL was assessed. (**f**) Pathway diagram: In 3T3-L1 adipocytes, both PIC and E2 activate PKA through GPER, leading to an increase in pHSL and promoting lipolysis from lipid droplets. These experiments were carried out three times, which confirmed the reproducibility of the results.

The expression levels of GPER protein were increased during differentiation of 3T3-L1 cells (Figure 3a). Using G15, a specific GPER antagonist, we investigated whether PIC-induced HSL phosphorylation was mediated by GPER (Figure 3b). G15 exhibits a high affinity for GPER and binds only minimally to ERα and ERβ (Ki >10 µM) [35]. The increased phosphorylation of HSL in PIC-treated cells was suppressed when cells were treated simultaneously with G15, the GPER inhibitor. These results suggest that PIC promotes HSL phosphorylation via GPER. Treatments with PIC and G15 did not alter the expression level of the GPER protein (Figure 3c). Therefore, PIC was shown to increase pHSL (Ser563) through the GPER signaling pathway, not increasing GPER expression.

Next, we examine the effect of estradiol (E2) on HSL phosphorylation (Figure 3d). Compared to cells without treatment (control), pHSL (Ser563) increased in those with E2 at physiological concentrations of 10^−8^ M and adjacent concentrations. Since ERα and ERβ function as nuclear receptors, and GPER mediates rapid signal transduction as a membrane receptor, the increase in pHSL (Ser563) after 1 h of short-term treatment with E2 suggests that HSL phosphorylation may be regulated through GPER rather than ERα or ERβ. The increase in HSL phosphorylation was shown to decrease when co-treated with the GPER-specific inhibitor G15 (Figure 3e).

These findings suggest that PIC acts as an agonist of GPER and activates HSL downstream of the GPER signal transduction pathway in adipocytes.

### 3.4. PIC Inhibits Akt Phosphorylation

**Figure 4:**
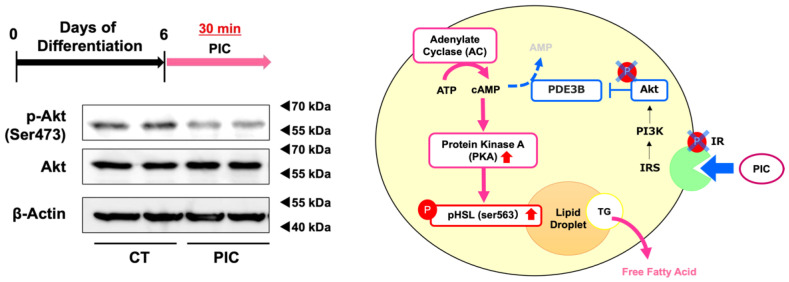
Piceatannol suppresses Akt phosphorylation in 3T3-L1 adipocytes. 3T3-L1 adipocytes were induced to differentiate and on day 6, cells were treated with PIC for 30 min and the protein expression level of phosphorylated Akt was evaluated. Pathway diagram: PIC may activate PKA by suppressing the phosphorylation of IR and Akt. These experiments were carried out three times, which confirmed the reproducibility of the results.

Kwon et al. showed that PIC inhibited the phosphorylation of the insulin receptor (IR)/insulin receptor substrate-1 (IRS-1)/Akt pathway in the early phase of adipogenesis by binding directly to IR [30]. We investigated whether PIC inhibits pAkt in ‘post-differentiated’ 3T3-L1 adipocytes. Treatment with PIC in 3T3-L1 adipocytes differentiated up to day 6 resulted in a decrease in Akt phosphorylation (Figure 4). PIC might increase cAMP levels by inhibiting the breakdown of cAMP to AMP through phosphodiesterase (PDE)-3B by reducing Akt phosphorylation. This could be considered a second pathway through which PIC increases pHSL (Ser563). It aligns well with the rapid signal transduction of the IR/Akt pathway, exhibiting a degree of consistency.

## 4. Discussion

The purpose of this study was to determine how PIC activates hormone-sensitive lipase (HSL) phosphorylation as observed in the white adipose tissue of OVX mice.

HSL was initially characterized as the hormonally regulated neutral lipase activity responsible for catalyzing the breakdown of triacylglycerols (TG) into fatty acids in adipose tissue. During lipolysis, HSL is translocated to the surface of LDs and participates, along with ATGL and monoacylglycerol lipase, in TG hydrolysis. Compared to other neutral lipases, HSL has an important feature: its regulation by hormones through reversible phosphorylation of sites located within the regulatory domain [36]. Therefore, we determined whether PIC activates HSL through the β-AR and PKA signaling pathways, which are well-known pathways for hormonal HSL activation. The results shown in Figure 2 indicate that PIC activation by PIC was dependent on PKA but not via β-AR.

In our previous study using OVX mice, the increase in pHSL (ser563) by PIC was a specific effect seen only in OVX mice, suggesting that the effect might be related to estrogen function due to its structural similarity to estradiol.

The loss of ovarian hormones leads to an increase in adiposity and insulin resistance, thereby increasing the risk of cardiovascular and metabolic diseases [4,5]. Based on the findings obtained from many animal models, such as OVX, Erα, and ERβ knockout mice, estrogen deficiency-induced obesity is believed to be due to reduced energy expenditure resulting from the loss of central nervous system signaling, reduced resting metabolic rate, and decreased spontaneous activity [6,37,38]. 17β-Estradiol inhibits visceral fat and induces the expression of genes specific for brown adipose tissue [39]. It is also well known that estrogen replacement therapy suppresses obesity [9,10].

Estrogen is involved in the expression of immune and female reproductive functions through estrogen receptors, ERα and ERβ, which regulate the gene expression as a classical nuclear receptor. Estrogen also regulates the expression of various genes related to lipid metabolism in adipocytes [22]. The addition of estradiol to adipocytes from abdominal fat adipocytes of human women decreased the protein expression of LPL and increased HSL [40]. However, GPER-mediated phosphorylation of HSL has not previously been reported.

Unlike classical estrogen receptors, GPER is a G protein-coupled receptor that orchestrates rapid responses through non-genomic actions. GPER is reported to be capable of mediating estrogenic activation through various signaling pathways, such as MAPKs [41,42], cAMP [24,42], and intracellular calcium [43]. GPER KO mice show obesity and insulin resistance [44]. In the induction of 3T3-L1 adipocyte differentiation, E2 has been reported to inhibit adipogenesis by perturbing mitotic clonal expansion through GPER signaling [45]. E2 and G1, a GPER agonist, were shown to enhance mitochondrial functions in inflamed adipocytes through a PKA-dependent mechanism [46]. However, no reports have indicated that GPER could be associated with the activation of lipolytic enzymes in adipocytes through GPER’s involvement in the PKA signaling pathway.

The present study revealed that PIC also phosphorylated HSL in 3T3-L1 differentiated adipocytes as shown in vivo, and that HSL phosphorylation was activated by the PKA pathway via GPER, but not β-AR. E2 also activated HSL phosphorylation. This is the first report to show that GPER, the non-genomic action of estrogen, may be implicated in lipolysis in adipose tissue through the phosphorylation of HSL by its downstream PKA pathway. PIC also has agonist activity against ERα and ERβ, but its activity has been suggested to be weaker than that of E2 [47,48]. Although the comparison of agonist activity against GPER with E2 was not evaluated in this study and requires further investigation, the fact that PIC was shown to have an anti-obesity effect via GPER suggests that PIC may be beneficial as an alternative treatment for obesity to ERT.

On the other hand, GPER stimulation is known to activate EGFR, leading to downstream activation of signaling molecules, such as ERK1 and ERK2 [41], and it is reported that ERK phosphorylates HSL (ser600) [49]. Although we did not examine the MAPK pathway in this study, the phosphorylation of HSL by the ERK pathway may also need to be further studied.

HSL activity is also known to be inhibited by insulin signaling. When insulin signaling induces phosphorylation of Akt, PDE-3B, an enzyme that catalyzes the breakdown of cAMP to AMP, becomes activated. This leads to a decrease in intracellular cAMP levels and subsequently reduces the activity of PKA, resulting in the inhibition of HSL phosphorylation. In the early stages of differentiation induction in 3T3-L1 adipocytes, PIC has been reported to dose-dependently inhibit the phosphorylation of the insulin receptor (IR)/insulin receptor substrate-1 (IRS-1)/Akt pathway. The same study demonstrated that PIC directly binds to IR and non-competitively inhibits IR kinase activity [30]. Our results shown in Figure 4 suggested that PIC also inhibits insulin signaling in the post-differentiation phase. In addition to these mechanisms described previously, the present results suggest that PIC reduces the size of adipocytes by facilitating the breakdown of TG accumulated in differentiated adipocytes.

Since PIC inhibits insulin signaling in adipocytes, caution is necessary when considering its actual therapeutic use, and it is crucial to verify its effects on other cell types. PIC has been shown to induce apoptosis in cancer cells [50]. In our investigation, higher concentrations (100 µM) of PIC exhibited toxicity in 3T3-L1 adipocytes. More research is required to assess the potential side effects associated with PIC, an aspect that needs future consideration.

Several reports on the inhibition of adipogenesis by PIC have been reported, including those using 3T3-L1 and stem cells derived from human visceral adipose tissue [51], and some reports have shown that PIC is more effective than RES [14]. RES, which is structurally similar to PIC, has been reported to be an agonist of GPER [31]. However, whether PIC acts directly as a regulatory agonist of GPER requires further investigation.

In conclusion, the findings presented here suggested that GPER and HSL activation were involved in fat accumulation in adipose tissue under estrogen deficiency. The results also showed that PIC acts as a phytoestrogen and promotes lipolysis by activating HSL. GPER-mediated activation of HSL may play an important role in estrogen-induced lipolysis of adipose tissue and inhibition of obesity.

## 5. Conclusions

In this study, we investigated the mechanisms by which PIC influences fat metabolism in 3T3-L1 adipocytes. Our findings revealed that PIC inhibits fat accumulation by enhancing HSL phosphorylation, independently of β-adrenergic receptors. Additionally, PIC’s effect through GPER emerged as a novel pathway for HSL activation. This newfound insight into the role in adipocytes may have profound implications for understanding obesity-related conditions. Furthermore, we discovered that PIC suppresses Akt phosphorylation in post-differentiated adipocytes. These findings demonstrate the multifaceted nature of the impact on adipocyte metabolism, revealing its potential as a therapeutic target for metabolic disorders.

## Data Availability

Data are available upon request to the corresponding author.

## Supplementary Materials

The following supporting information can be downloaded at: https://www.mdpi.com/article/10.3390/nu16010038/s1, Figure S1: Oil Red O staining on day 6 in 3T3-L1 cells.
