# Supplementary material for: GPER/PKA-Dependent Enhancement of Hormone-Sensitive Lipase Phosphorylation in 3T3-L1 Adipocytes by Piceatannol
Source: Nutrients. 2023 Dec 21;16(1):38. doi: 10.3390/nu16010038 (PMC10781143; doi:10.3390/nu16010038)
Supplement: Supplementary file 1 [file nutrients-16-00038-s001.zip › nutrients-2704301-supplementary.pdf]

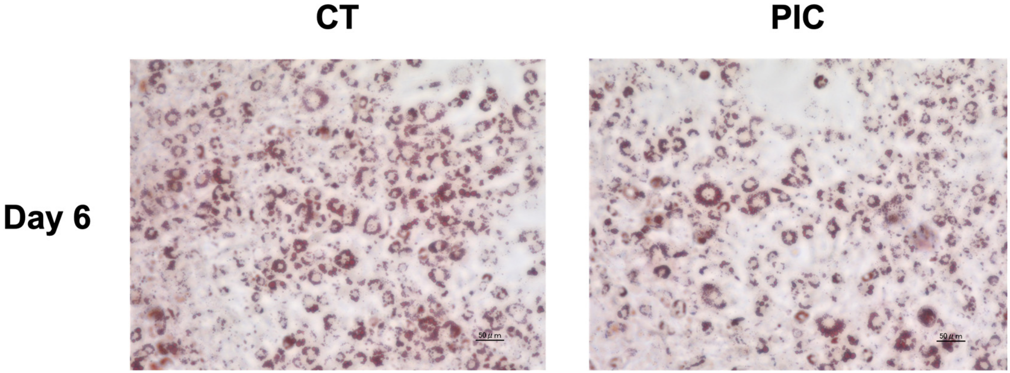

To induce adipocyte differentiation, 3T3-L1 cells were treated with insulin, IBMX, and dexamethasone. PIC (25 μM) was added to cells every other day, commencing at the beginning of differentiation and continuing for a period of 6 days. Oil Red O staining was used to visualize and evaluate lipid droplets in 3T3-L1 cells.
